# Athletic Identity and Sport Injury Processes and Outcomes in Young Athletes: A Supplemental Narrative Review

**DOI:** 10.3390/jfmk9040191

**Published:** 2024-10-09

**Authors:** Britton W. Brewer, Hailey A. Chatterton

**Affiliations:** Department of Psychology, Springfield College, Springfield, MA 01109, USA; hchatterton@springfieldcollege.edu

**Keywords:** adolescent, rehabilitation, self-identity, youth

## Abstract

**Background**: Identity formation, a primary developmental task of adolescence, may be particularly relevant to another commonly occurring event for young athletes—sport injury. Relationships between a subdimension of self-identity—athletic identity—and sport injury processes and outcomes have been documented in the general athlete population. The purpose of this supplemental narrative review is to explore the potential role of athletic identity in the risk of injury occurrence and responses to and consequences of injury among young athletes. **Methods**: Studies on athletic identity in relation to sport injury, with a focus on young athletes, were extracted from a recent scoping review and identified through an updated literature search from April 2020 through June 2024. A total of 23 studies were examined. **Results**: Across the studies reviewed, high levels of athletic identity were associated with a reluctance to report injury-related symptoms, a tendency to endorse attitudes and behaviors reflecting a willingness to play through pain and injury, intensified physical and psychological symptoms after injury, a disposition toward over-adhering to rehabilitation, high levels of postinjury coping skills, and better functional and return-to-sport outcomes after injury among young athletes. **Conclusions**: Athletic identity may, therefore, be a source of both strength and vulnerability in young athletes in terms of sport injury processes and outcomes.

## 1. Introduction

Growth and development are primary themes of adolescence. In the psychosocial realm, a primary developmental task of adolescence is identity formation [1]. Identity (also known as self-identity) is “a clearly delineated self-definition…comprised of those goals, values, and beliefs which the person finds personally expressive, and to which he or she is unequivocally committed” [2] (p. 6). Identity has been described as a socially constructed mental construct that guides and organizes the processing of self-related information and influences behavior [3,4,5,6]. Further, identity is considered to be multidimensional, with each individual’s identity composed of a unique, idiosyncratic combination of roles and personal attributes that are deemed to be self-defining and vary in prominence [7]. Marcia [8] proposed that when people firmly commit to an identity without first having explored meaningful alternatives, they are in a state of identity foreclosure.

Identity and identity formation are relevant to both the general population and to specific populations, including athletes. By virtue of their involvement in sport, some individuals develop a strong connection with the athlete role as a source of self-definition and self-worth. This connection has been labeled “athletic identity” [9]. From a developmental standpoint, athletic identity among participants in competitive sport tends to increase from late childhood to adolescence and remain stable into early adulthood [10]. When young athletes commit to the athlete role as a primary source of self-identification without having engaged in exploration of other identities, roles, and ideologies, they develop what has been termed athletic identity foreclosure [11]. Consistent with perspectives on the self and identity in general [3,4,5,6], sport participants’ levels of athletic identity have important implications for their cognitive, emotional, and behavioral responses to sport-related events [9]. There are documented associations between athletic identity and both “positive” (i.e., desired) and “negative” (i.e., “undesired”) motivational and emotional factors [12] and variables pertaining to disparate phenomena such as burnout, career development, substance use, and psychological adjustment to sport transitions such as sport career termination and sport injury [13].

The potential role of athletic identity in relation to the latter phenomenon—sport injury—has been subjected to empirical investigation for more than three decades. In a scoping review of the quantitative literature on athletic identity in association with sport injury processes and outcomes, it was found that athletic identity was positively related to injury-related factors such as postinjury depressive symptoms, over-adherence to injury rehabilitation, and functional rehabilitation outcomes [14]. Further, in a systematic review of mental health consequences of musculoskeletal injury in pediatric athletes, it was concluded that “stronger athlete identity is a risk factor for the development of depressive symptoms” [15] (p. 1). Given the high incidence of sport injury among young athletes [16], the centrality of identity development during adolescence [1], and documented associations between athletic identity and sport injury processes and outcomes for athletes in general [14], it is important to examine the potential role of athletic identity in the occurrence of, responses to, and rehabilitation of sport injuries in young athletes. Consequently, the purpose of this report is to summarize content from the scoping review of Renton et al. [14] that is relevant to young athletes and provide an update that features findings from more recent studies of athletic identity in the context of sport injury that were not included in the scoping review.

## 2. Supplemental Narrative Review Method

### 2.1. Search Process

For the purposes of this project, “young athletes” were defined as those from 10 to 24 years of age, which is consistent with the definitions of “adolescence” provided by the American Academy of Pediatrics [17] and “young people” provided by the World Health Organization [18]), respectively. Relevant studies were identified through two primary methods. First, investigations included in the review by Renton et al. [14] that featured young athlete samples were selected. This approach yielded 11 studies. Second, guided by the search strategy used by Renton et al., the APA PsycInfo, MEDLINE Complete, PubMed, Rehabilitation and Sports Medicine Source, and SPORTDiscus with Full-Text databases were searched in July 2024 with the following terms: Search term (2020 or later): (athlete OR sports OR sport OR athletic) AND (identity) AND (injury). Searches of the five databases yielded 197 nonredundant articles that were not among those included in Renton et al.’s [14] review.

### 2.2. Inclusion and Exclusion Criteria

Criteria for inclusion and exclusion were guided by those used by Renton et al. [14]. To be included in the supplemental narrative review, studies needed to feature a young athlete sample of any playing status, at least one group of participants with a sport-related injury that prevented sport participation, and a quantitative self-report assessment of athletic identity. Samples involving participants of various ages were considered young athlete samples if the mean age of participants fell within the ages designated as corresponding to “adolescence” [17] and “young people” [18]. Articles that reported qualitative research, theses or dissertations, consensus statements, literature reviews (i.e., systematic, scoping, narrative), conference proceedings or abstracts, and papers that were not published in the English language or did not feature self-report assessment of athletic identity were excluded from the supplemental narrative review.

### 2.3. Data Extraction

For the included studies that were derived from the previous review [14], data were extracted from Table 2 (pp. 7–18) of Renton et al.’s [14] article. For the included studies that were derived from the updated search of the literature, data were derived directly from the articles in which the studies were described. For each of the included studies, the data that were extracted included a description of the sample (i.e., sample size, sex, age, sport, level of sport involvement), type of injury, study design, and key findings pertaining to athletic identity.

## 3. Supplemental Narrative Review Results

In addition to the 11 studies included in Renton et al.’s [14] review, the search of the more recent literature yielded 12 studies. Of the 23 total studies included in the supplemental narrative review, 9 were related to the risk of occurrence of sport injury and 15 were related to responses to or consequences of sport injury (1 study was related to both risk and responses/consequences).

### 3.1. Athletic Identity and Risk for Occurrence of Sport Injury

As summarized in Table 1, a growing body of research has documented associations between athletic identity and both the occurrence of sport injuries and factors putatively related to the occurrence of sport injuries in young athletes. McKay et al. [19] found that low levels of athletic identity were associated with an elevated risk of an initial injury, whereas high levels of athletic identity were associated with elevated risk of subsequent injury among elite youth ice hockey players. In contrast to the findings of McKay et al. [19], Petrie et al. [20] found that athletic identity was not predictive of time lost from sport participation due to injury in a small sample of intercollegiate American football (gridiron) players. Similarly, Johansson et al. [21] found that athletic identity was not significantly related to the occurrence of shoulder overuse injuries in competitive adolescent tennis players.

Most of the remaining studies in this category involved examination of associations between athletic identity and behaviors or attitudes that presumably increase the risk of the occurrence of sport injury. Three studies were identified in which the relationship between athletic identity and the willingness or intention to report concussion symptoms was investigated. Kroshus et al. [22] found that although athletic identity was not directly related to the failure to report concussion symptoms in male intercollegiate ice hockey players, athletic identity moderated the association between perceived reporting norms for “most athletes” and symptom non-reporting, such that “The likelihood of under-reporting is increased when athletic identity is strong and perceived reporting norms are less safe” (p. 100). Martin et al. [23] documented a significant negative zero-order correlation between athletic identity and the willingness to report concussion symptoms in a sample of high school and college club sport athletes, but the association was no longer significant when the contributions of other relevant predictors (i.e., gender, age, diagnosed with concussion, concussion knowledge, the perceived seriousness of concussion, concern about concussion impacting future health, harmonious passion, and obsessive passion) were included in the analysis. Most recently, Baer et al. [24] reported a weak statistically significant inverse relationship between athletic identity and the intention to report concussion symptoms in intercollegiate athletes that was no longer significant when reasoned action approach [25] variables were entered into the predictive equation.

Associations between athletic identity and behaviors and attitudes pertaining to playing through pain and injury were assessed in three studies that were included in the review. Weinberg et al. [26] found that college intramural athletes with high athletic identity scores were more likely to report having positive attitudes toward and behaviors involving playing through pain and injury than those with low athletic identity scores. In an investigation of intercollegiate and college club sport athletes, Monaco et al. [27] documented that athletic identity was “positively associated with the number of concussions that participants with a history of one or more concussions reported would prompt them to retire from their primary sport” (p. 1). Consistent with the results of Weinberg et al. [26] and Monaco et al. [27], Johansson et al. [21] found that adolescent tennis players with a strong athletic identity were more likely to report playing through pain than those with a weaker athletic identity.

One other study in which associations between athletic identity and factors potentially associated with risk for sport injury occurrence were examined was included in the review. In a study of young athletes with ACL tears, Padaki et al. [28] found that single-sport participants scored higher for athletic identity than multisport participants.

**Table 1:** Studies examining athletic identity and factors related to risk of sport injury occurrence in young athletes.

Study	Participants (Sex, Age)/ Research Design	Relevant Findings
McKay et al. [19]	316 male elite youth ice hockey players; 15 years (median); longitudinal	low levels of athletic identity were associated with elevated risk of initial injury; high levels of athletic identity were associated with elevated risk for subsequent injury
Weinberg et al. [26]	68 male and 62 female college intramural basketball players; 20.03 (1.60) years; cross-sectional	compared to low-athletic-identity participants, high-athletic-identity participants were more likely to report positive attitudes toward and behaviors involving playing through pain and injury
Petrie et al. [20]	26 male intercollegiate American football players; 20.08 (1.46) years; longitudinal	athletic identity was not predictive of time lost from sport participation due to injury
Kroshus et al. [22]	146 male intercollegiate ice hockey players; longitudinal	athletic identity moderated the relationship between perceived reporting norms and concussion symptom non-reporting
Padaki et al. [28]	12 female and 12 male young athletes with ACL tears; 14.5 (2.7) years; cross-sectional	single-sport participants had higher athletic identity scores than multisport participants
Monaco et al. [27]	199 male and 195 female intercollegiate and college club sport athletes; 19.77 (1.57) years; cross-sectional	for participants with a concussion history, athletic identity was positively associated with an estimate of how many concussions they would have to sustain before retiring from their primary sport
Johansson et al. [21]	155 male and 114 female competitive adolescent tennis players; 14.5 (2.0) years; longitudinal	athletic identity was not significantly related to the occurrence of shoulder overuse injuries, but was positively associated with a tendency to report playing through pain
Martin et al. [23]	203 male and 119 female high school and club sport athletes; 15.7 (1.34) years; cross-sectional	there was significant negative zero-order correlation between athletic identity and the willingness to report concussion symptoms; the relationship was nonsignificant in a full regression model
Baer et al. [24]	1712 female, 927 male, and 10 unspecified-gender intercollegiate athletes; 20.02 (1.36) years; cross-sectional	there was a weak statistically significant inverse relationship between athletic identity and intention to report concussion symptoms; the relationship was no longer significant in a full regression model

### 3.2. Athletic Identity and Responses to or Consequences of Sport Injury

As shown in Table 2, the majority of the quantitative studies of athletic identity in association with sport injury processes and outcomes in young athletes pertain to responses to or consequences of sport injury. Relations between athletic identity and psychological or physical outcomes after sport injury were examined in seven studies. In an investigation of intercollegiate American football players [29], athletic identity was positively correlated with depressed mood in participants who were injured and negatively correlated with depressed mood in participants who were not injured. Similarly, Manuel et al. [30] reported that athletic identity was positively associated with depression scores among young athletes with musculoskeletal injuries that prevented sport participation for at least three weeks. Although O’Rourke et al. [31] found that athletic identity was positively associated with the severity of postconcussion symptoms in young athletes, Padaki et al. [28] found no differences in posttraumatic symptoms after ACL injury as a function of athletic identity level. In contrast to the findings of Brewer [29], Manuel et al. [30], O’Rourke et al. [31], and Padaki et al. [28], Ohji et al. [32] found that young athletes who returned to sport after anterior cruciate ligament (ACL) reconstruction at the same competitive level as before their injuries reported higher athletic identity scores than young athletes who did not return to sport at the same competitive level. Consistent with the results of Ohji et al. [32], young athletes with high athletic identity scores reported higher levels of functional activity both before ACL reconstruction [33] and one year after ACL reconstruction [34]. In a cross-sectional study of young athletes who had sustained an ankle sprain injury 3 to 15 years earlier, Owoeye et al. [35] found that athletic identity was not significantly associated with ankle-related symptoms and functioning.

The relationship between athletic identity and over-adherent behavior in sport injury rehabilitation was explored in two studies, both of which were included in the scoping review of Renton et al. [14]. In a pair of studies, Podlog et al. [36] found that athletic identity was positively associated with self-reported tendencies to attempt to expedite rehabilitation and ignore practitioner recommendations among young athletes with injuries. Similarly, Hilliard et al. [37] reported positive associations between athletic identity and over-adherence to rehabilitation, attempts to expedite rehabilitation, and willingness to ignore practitioner recommendations.

Four studies identified in the updated search of the literature documented associations between athletic identity and other psychological factors related to coping during sport injury rehabilitation. Ohji et al. [38] found that athletic identity was not significantly correlated with psychological readiness to return to sport assessed prior to the ACL surgery of young athletes. In contrast, Tatsumi [39] reported that among young athletes who had experienced an injury, athletic identity was positively associated with the coping strategies of positive reappraisal and suppression of expression of negative emotions. Although positive reappraisal and suppression of expression were directly related to each other, the former was deemed an adaptive coping strategy and the latter was considered potentially maladaptive. Ferman et al. [40] found that for adolescent athletes with musculoskeletal injuries, athletic identity was inversely related to fear avoidance perceptions during a return to sport. Additionally, McGinley et al. [34] showed that young athletes who scored highly in terms of athletic identity had higher self-reported coping ability than young athletes with a low athletic identity score one year after ACL reconstruction.

Finally, there was a pair of recent studies in which athletic identity was examined in the context of sport injury in ways unlike those in other investigations. Werner et al. [41] reported no significant differences in athletic identity across young athletes with various injury profiles (i.e., chronically injured and receiving medical treatment, seeking little medical treatment or rehabilitation, and infrequently but severely injured and receiving medical treatment) and McGinley et al. [33] found that the athletic identity of young athletes decreased significantly in the first year after ACL reconstruction.

**Table 2:** Studies examining athletic identity and responses to and consequences of sport injury in young athletes.

Study	Participants (Sex, Age)/ Research Design	Relevant Findings
Brewer [29]	90 male intercollegiate American football players; cross-sectional	athletic identity was positively correlated with depressed mood for players who were injured and negatively correlated with depressed mood for players who were not injured
Manuel et al. [30]	28 female and 20 male young athletes; 16–18 years (range); cross-sectional	athletic identity was positively associated with depression
Podlog et al. [36]	61 male and 57 female young athletes, 15.97 (1.41) years (Study 1); 62 male and 43 female intercollegiate athletes (Study 2); both cross-sectional	athletic identity was positively associated with self-reported tendencies to attempt to expedite rehabilitation and ignore practitioner recommendations
Hilliard et al. [37]	51 male and 28 female intercollegiate athletes; 19.96 (1.56) years; cross-sectional	athletic identity was positively associated with over-adherence to rehabilitation, attempts to expedite rehabilitation, and willingness to ignore practitioner recommendations
O’Rourke et al. [31]	27 female and 24 male young athletes in various sports; 14.53 (1.85) years; longitudinal	athletic identity was positively associated with severity of postconcussion symptoms
Padaki et al. [28]	12 female and 12 male young athletes with ACL tears; cross-sectional	posttraumatic symptoms did not differ as a function of athletic identity level
Ohji et al. [32]	22 male and 17 female athletes who had ACL reconstruction; ≤23 years (median); cross-sectional	participants who returned to sport after ACL reconstruction at the same competitive level as before their injuries reported higher athletic identity than those who did not
McGinley et al. [33]	115 female and 111 male young athletes scheduled for ACL reconstruction; 15.9 (2.1) years; cross-sectional	participants with high athletic identity scores reported higher levels of functional activity
Owoeye et al. [35]	66 female and 20 male young athletes; 23 years (median); cross-sectional	athletic identity was not significantly associated with ankle-related symptoms and functioning
Ohji et al. [38]	59 male and 46 female athletes scheduled for ACL reconstruction; 20.0 (9.0) years; cross-sectional	athletic identity was not significantly correlated with psychological readiness to return to sport assessed prior to the ACL surgery
Tatsumi [39]	59 male and 46 female young athletes with prior injuries; 20.27 (1.02) years; retrospective	athletic identity was positively associated with coping strategies such as positive reappraisal and suppression of negative emotions
Werner et al. [41]	115 female, 95 male, and 3 non-binary athletes varying in injury status; 22.77 (3.57) years; cross-sectional	athletic identity was not significantly different across various injury profiles
Ferman et al. [40]	26 female and 24 male young athletes in return-to-sport phase of musculoskeletal sport injury rehabilitation; 16.8 years; cross-sectional	athletic identity was inversely related to fear avoidance perceptions
McGinley et al. [34]	45 female and 42 male young athletes who had ACL reconstruction; 15.3 (1.8) years; longitudinal	participants who scored highly in terms of athletic identity reported higher levels of functional and coping ability than those with a low athletic identity score one year after ACL reconstruction; athletic identity decreased significantly in the first year after ACL reconstruction

## 4. Discussion

The purpose of this supplemental narrative review was to examine findings from quantitative studies in which athletic identity was investigated in the context of sport injury processes and outcomes in young athletes. Relevant studies were identified both from a scoping review on the topic among athletes of all ages [14] and an updated search of the literature published after the end of the period covered in the scoping review. From a purely descriptive standpoint, it is noteworthy that there were 9 studies that met the selection criteria that were published from 1946 to early 2020 and 12 studies that met the selection criteria published from early 2020 to June 2024, suggesting that awareness of the relevance of athletic identity to sport injury processes and outcomes of athletes in general, and young athletes in particular, has increased among investigators.

Although minimal support was found for the claim that athletic identity is related to the occurrence of sport injuries in young athletes, there were more consistent findings pertaining to the relationship between athletic identity and attitudes and behaviors that may increase the risk of sport injury occurrence in young athletes. Associations between athletic identity and willingness or intent to report concussion symptoms tended to be weak and positive, indicating a possible reluctance of young athletes who scored highly in athletic identity to seek medical assistance for mild traumatic brain injuries sustained in the course of sport participation. Such a trend is consistent with research suggesting that a strong athletic identity is a barrier to seeking help for mental health issues [42]. Further, across three studies included in the review, high levels of athletic identity were consistently associated with attitudes and behaviors involving an inclination toward playing through pain and injury, a practice that embodies the constellation of norms and values known as the “sport ethic” [43] and presumably places athletes at elevated risk of further pain and injury.

Associations between athletic identity and postinjury variables (i.e., responses to and consequences of sport injury) were more mixed than those between athletic identity and variables related to injury risk. High levels of athletic identity have been accompanied by both favorable outcomes (i.e., better functional and return-to-sport status) and unfavorable outcomes (i.e., severity of depression and concussion symptoms) in young athletes. Just as a positive relationship between athletic identity and adherence to sport injury rehabilitation has been documented among young athletes in previous research [44], two studies in the current review showed positive relationships between athletic identity and *over*-adherence to sport injury rehabilitation in young athletes. In contrast, positive associations between athletic identity and adaptive coping processes were found in multiple studies involving young athletes with injuries.

Although a strength of the studies examined in the supplemental narrative review is that they cover an array of injury-related processes and outcomes, and feature participants that vary widely in terms of sport and injury type, it is important to note the limitations of the current review and the literature on which it is based. First, in line with the scoping review of Renton et al. [14], only studies in which athletic identity and sport injury processes and outcomes were assessed quantitatively were considered. Examination of the large and growing body of studies with qualitative data may shed greater light on the centrality of athletic identity in relation to sport injury phenomena. For example, in a qualitative study of elite adolescent athletes with injuries by Von Rosen et al. [45], the overarching theme of participants’ responses was “Injury as a threat to the identity of a young athlete” (p. 7). Similarly, in a systematic review of the qualitative literature on elite athletes’ responses to sport injury [46], it was concluded that identity loss was a commonly reported aftereffect of injury. Second, even with the expansion of research on athletic identity in the context of sport injury that has occurred since the publication of the scoping review of Renton et al. [14], the number of studies examining any particular sport injury process or outcome in association with athletic identity in young athletes is small. Confidence in the reliability of the findings of reviews such as the current one will grow with a larger population of studies on the topic. Third, the majority of the studies on athletic identity in relation to sport injury processes and outcomes in young athletes are cross-sectional. As with sport injury processes and outcomes, athletic identity is dynamic, especially during adolescence [13], and, therefore, requires longitudinal designs to capture changes in the construct and its relations to other constructs that may occur over time. Fourth, as noted by Renton et al. [14] and Loftin et al. [47], research on athletic identity in the context of sport injury processes and outcomes has not consistently been guided and/or interpreted by theoretical models. The absence of a unifying theory or set of theories has given this body of research a patchwork appearance and has made it difficult to interpret disparate findings. In light of the limitations of the current review and the studies on which it is founded, it can safely be concluded that the knowledge gap pertaining to the role of athletic identity across the full range of sport injury processes and outcomes in young athletes is wide, providing investigators with an abundance of opportunities to contribute substantively to the literature.

## 5. Future Directions

A primary direction for future inquiry on athletic identity in association with sport injury processes and outcomes among young athletes is to address the limitations noted for both the current review and the literature on which it is based. Creating a larger volume of research on the topic, reviewing and conducting studies with qualitative and mixed-methods approaches, implementing prospective longitudinal and experimental research designs, and using theory to inform both the research questions that are asked and how the findings are interpreted are sure to elevate the discourse on the potential contributions of athletic identity to the occurrence of and responses to sport injury in young athletes. Furthermore, given the wide age range of individuals considered as “young athletes” in the current review and the developmental changes that occur physically and psychosocially over the course of adolescence, more refined analyses of associations between athletic identity and sport injury processes and outcomes by age are needed. In addition, given the threats to body integrity posed by sport injury and the potential of environmental influences to mitigate or exacerbate the impact of such threats, inclusion of measures of body image and environmental factors in investigations of the relationship between athletic identity and sport injury processes and outcomes may also prove fruitful.

Another task for future research is to examine some of the relationships documented in the current review from a more nuanced perspective. For example, the findings that young athletes with high levels of athletic identity are more likely to return to the same competitive level after ACL reconstruction [32] and experience fewer fear avoidance perceptions when returning to sport after injury [40] than young athletes with a low athletic identity score can be interpreted as indicating that athletic identity is a motivating factor that inspires a positive mindset towards [39] and adherence to rehabilitation [44] and reduces apprehension when resuming sport participation after rehabilitation is complete. Conversely, the very same findings can be interpreted as reflecting that athletic identity is a *hypermotivating* factor that prompts young athletes to play through pain and injury [21,26,27], neglect to report symptoms of injury [22,23,24], over-adhere to rehabilitation [36,37], suppress expression of negative emotions [39], and fail to engage in a rational assessment of the risks associated with returning to sport after a serious injury [40].

**Figure 1:**
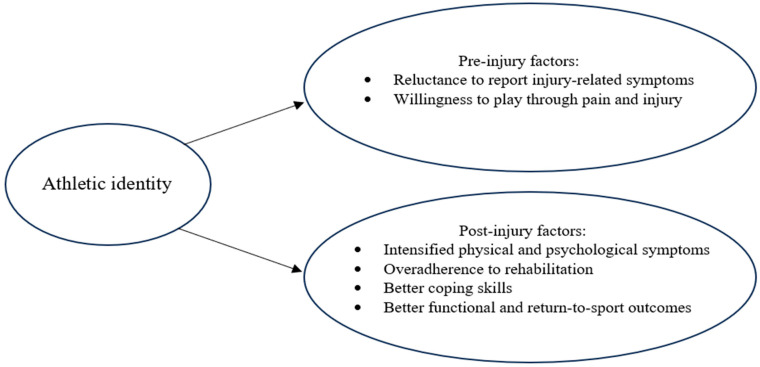
Pre-injury and postinjury correlates of athletic identity in young athletes.

Teasing apart adaptive and maladaptive contributions of athletic identity to sport injury rehabilitation processes and outcomes is essential to guiding application of knowledge gleaned from this line of inquiry. Based on the existing body of research, it has been recommended that the athletic identity of patients should be considered in clinical decision-making and treatment planning in sports health care [40,47,48,49,50]. To this end, the graphic summary of the results of the current review depicted in Figure 1 may be useful for helping sports health care practitioners, coaches, parents, and young athletes gain a nuanced understanding of the potential role that athletic identity may play in the success of preventive and rehabilitative interventions. If future research more definitively demonstrates a causal role of athletic identity in precipitating maladaptive sport injury processes and outcomes, it would be prudent to heed the stark advice of Nyland and Pyle [49] that “Self-identity should originate from a variety of sources and roles; it should not be solely dependent on athletics” (p. e292) and implement interventions that broaden the self-identities of young athletes [51,52]. Such interventions, which could be delivered proactively (i.e., prior to injury) or after injury occurrence [47], could also have salubrious collateral effects on academic, burnout, and career development outcomes in young athletes [53].

An additional potential application of athletic identity in the sport injury domain involves harnessing high levels of athletic identity to encourage engagement in injury-preventive activities. The same motivation that prompts young athletes with high athletic identity scores to adhere to postinjury rehabilitation programs to a greater extent than young athletes low in athletic identity [44] can conceivably be tapped to stimulate adherence to injury prevention programs and avoidance of behaviors that increase risk for injury. Empirical examination of this possibility seems warranted.

## 6. Conclusions

It has long been recognized that strong self-identification as an athlete has the potential to confer both strength and vulnerability within sport participants [9]. Based on the findings of the current review, such seems to be the case with respect to sport injury processes and outcomes for young athletes. Vulnerability is more salient than strength in terms of the risk of sport injury occurrence, as high levels of athletic identity are associated with a reluctance to report injury-related symptoms and a tendency to endorse attitudes and behaviors that reflect a willingness to play through pain and injury. Vulnerability is also apparent after the occurrence of sport injury, with high levels of athletic identity corresponding to intensified physical and psychological symptoms and a disposition toward over-adhering to rehabilitation. Evidence of potential strength associated with self-identifying strongly as an athlete after the occurrence of injury can be found in the form of elevated motivation to engage vigorously in rehabilitation activities, higher levels of coping skills, and better functional and return-to-sport outcomes.

Because of the small size and mostly preliminary nature of the research base, these conclusions are offered cautiously and as hypotheses for further inquiry on the topic. A more thorough understanding of the contributions of athletic identity to sport injury processes and outcomes has the potential to inform the practice of professionals who work with young athletes and, ultimately, bolster efforts to prevent the occurrence of sport injuries and enhance the rehabilitation of athletes who experience injuries.

## Data Availability

No new data were created or collected for this review.
